# Working with the inner critic in patients with depression using chairwork: a pilot study

**DOI:** 10.3389/fpsyt.2024.1397925

**Published:** 2024-07-01

**Authors:** Julia Kroener, Jacqueline Mahler, Zrinka Sosic-Vasic

**Affiliations:** ^1^ Christophsbad-Academy for Psychotherapy, Department of Applied Psychotherapy and Psychiatry, Christophsbad Goeppingen, Goeppingen, Germany; ^2^ Medical Department, University of Ulm, Ulm, Germany

**Keywords:** CBT, cognitive-behavioral therapy, depression, short-intervention, self-criticism, chairwork, emotion-focused therapy, self-compassion

## Abstract

**Introduction:**

Individuals diagnosed with depression frequently experience self-criticism, leading to considerable psychological distress. Despite the availability of cognitive-behavioral treatments, a notable proportion of patients indicate that they solely experience cognitive improvements, without the corresponding emotional changes, following therapy. As a result, their psychological symptoms persist. Interventions that specifically target emotional experiencing, such as the chairwork technique, are exclusively included within long-term therapeutic procedures. Hence, the objective of this study is to assess the efficacy as well as the acceptability, feasibility, and safety of a brief intervention utilizing emotion-focused chairwork to treat self-criticism in individuals diagnosed with depression.

**Methods:**

A pre-post A-B design with two post-treatment assessments (one week- and one month post-intervention) was implemented. Seven patients received three sessions of manualized emotion focused chairwork. Symptomatic change was evaluated using the Beck Depression Inventory II (BDI-II), the emotion regulation questionnaire (SEK-27), the Forms of Self-Criticizing/Reassuring Scale (FSCRS), the Self-Compassion Scale (SCS-D), as well as the Rosenberg Self-Esteem Scale (RSES). Patient satisfaction was evaluated using a self-developed questionnaire. Safety was assessed by the Beck Suicidality Inventory (BSI).

**Results:**

There was a significant improvement in depressive symptoms and self-compassion at both follow-up assessment time-points. Moreover, emotion regulation as well as self-esteem improved significantly. Self-criticizing decreased significantly, while self-reassuring increased. Patients were very satisfied with the intervention. Intervention safety was given at all time-points. There were no drop-outs.

**Conclusion:**

The implemented chairwork short-intervention is a feasible and safe therapeutic technique. The treatment was highly accepted revealing significant symptomatic improvements. Large-scale randomized controlled trials (RCTs) are necessary to investigate the treatment’s effectiveness.

## 1 Introduction

Individuals diagnosed with depression commonly experience symptoms such as reduced interest in activities, disrupted sleep patterns, feelings of sadness, a sense of worthlessness, low self-esteem, and suicidal thoughts (1). In general, depression is characterized by a pervasive feeling of global distress, which leads to an inability to accurately discriminate and identify emotions (2, 3). Dysfunctional mood congruent self-critical cognitions and negative core beliefs further foster unspecific negative emotional states (2, 3). The application of Beck’s 5-column thought record (4) or the utilization of Socratic questioning (5) are fundamental approaches within cognitive-behavioral therapy (CBT) when engaging with individuals experiencing depression, with the aim of altering maladaptive cognitions and underlying core beliefs. Nevertheless, the regular application of these cognitive processes often results in cognitive transformation, while the corresponding emotional transformation does not occur simultaneously (referred to as “heart-head” lag 4, 6, 7). Consequently, a considerable proportion of patients do not respond to the aforementioned cognitive strategies (8). One commonly employed yet insufficiently investigated technique in psychotherapy to address this challenge is chairwork.

Emotion-focused chairwork originates within psychodrama, where it has been invented by Moreno (9). Later on, it has been extended and elaborated by Pearls (10), the founder of gestalt therapy. Ever since, chairwork has been adapted and implemented within various psychotherapeutic stances, such as emotion focused therapy (11) or schema-therapy (e.g., 12, 13), both of which originate within the third wave of cognitive behavioral therapy. Overall, chairwork techniques follow a dialogical structure, signifying that different parts of the problem (outside and inside oneself) are being placed-, and reenacted on various chairs (e.g., 14–16). This experiential technique follows three overarching principles: (1) multiplicity (i.e., the self is multifaceted, and relevant parts of the self can be differentiated through placement in separate chairs); (2) embodiment and personification (i.e., the self-parts can be made “human-like” through enactment by the patient, in order to facilitate exchange of information), and (3) dialogue (i.e., encouragement of the self-parts to speak to one another, to the patient, or the therapist, in order to ameliorate distress and/or resolve conflicts; 15, p.3).

Chairwork has been utilized in the context of Cognitive Behavioral Therapy (CBT) to modify core beliefs (4). This intervention is particularly utilized when changes on a cognitive level take place without concomitant emotional alterations. Changes on an emotional level are particularly important, as past research has shown that improving emotional processing in individuals with depression leads to better therapeutic outcomes (17–19). In addition, chairwork has been employed as a technique to solve ambivalence (e.g., 7, 20, 21), modify traumatic childhood memories (e.g., 22, 23), evoke intense emotions to facilitate exposure and habituation (e.g., 23, 24), or decrease experiential avoidance (e.g., 6, 25). Furthermore, chairwork has been used within the context of third-wave CBT to address self-criticism (26–28) and early maladaptive schemas (12). Addressing self-criticism during psychotherapeutic treatment holds significant value for several reasons. Firstly, self-criticism inherits a pivotal role in the manifestation of psychological distress, and is secondly prevalent across a wide range of clinical disorders. Lastly, past studies suggest that self-criticism is linked to poor treatment outcomes (29, 30).

Empirical evidence pertaining to the efficacy of chairwork as an independent therapeutic intervention displays a relatively modest yet encouraging body of studies suggesting its efficacy. For instance, de Oliveira et al. (30–32) demonstrated that the implementation of trial-based chairwork during a single therapeutic session has a noteworthy effect on diminishing negative core beliefs and associated emotions. Moreover, there was a notable decrease in self-criticism after the intervention. Furthermore, Shahar et al. (29) conducted a pilot study to examine the efficacy of the two-chair technique in diminishing self-criticism and promoting self-compassion among a subclinical sample of individuals experiencing symptoms of depression and anxiety. Results revealed medium to high effect sizes, which persisted at the follow-up assessment conducted six months after treatment termination. Furthermore, the same two-chair technique can effectively reduce global distress, rumination, as well as thought suppression (33, 34). Moreover, based on initial findings, a comparison of short-term therapies utilizing standardized cognitive restructuring and chairwork suggests that chairwork has greater efficacy than cognitive restructuring in reducing fears of being evaluated among individuals experiencing social anxiety (30, 32, 35). Lastly, Stiegler et al. (36) investigated the efficacy of five sessions implementing the two-chair dialogue in alleviating symptoms of depression and anxiety in individuals on paid sick leave. The multiple baseline study displayed statistically significant results, indicating a notable reduction in depressive and anxiety symptoms (36).

Nevertheless, the above described preliminary results on the efficacy of chairwork exhibit significant limitations. First, the samples were partially comprised out of convenience samples (34) and subclinical samples (29). Second, studies, which did include clinical samples, did not conduct structured diagnostic interviews to confirm the clinical diagnoses (32, 36). Third, looking at the implemented study designs, naturalistic designs (32), quasi-experimental designs (29, 34), and an additive component design (36) were employed. There was solely one study (35), which did conduct a randomized controlled trial (RCT). However, this study also included other CBT techniques along with the conduct of chairwork interventions. Hence, the aforementioned studies exhibit limitations in terms of their generalizability and reliability. Consequently, it becomes imperative to conduct further clinical trials that encompass patients with confirmed clinical diagnoses and sufficiently large sample sizes. Such trials would allow for more precise inferences regarding the efficacy and safety of the chairwork technique.

## 2 Objective

The primary aim of this study is to assess the efficacy, feasibility, acceptability, and safety of a short-intervention utilizing CBT based chairwork to treat self-criticism in individuals diagnosed with depression. We hypothesize that the implementation of a standardized three-session chairwork intervention has the potential to effectively diminish self-criticism and augment self-compassion in individuals diagnosed with depression within an outpatient setting. Furthermore, we postulate that conducting three sessions of chairwork will result in a decrease in depressive symptoms, while simultaneously promoting adaptive emotion regulation and enhancing self-esteem. Finally, we hypothesize that the therapeutic outcomes post-intervention will remain stable during the one-month follow-up period.

## 3 Materials and methods

### 3.1 Design

A pre-post A-B design was utilized, incorporating two post-treatment evaluations. This approach was chosen to determine the feasibility of implementing three sessions of chairwork to reduce self-criticism in patients diagnosed with depression. The study was performed as a single-arm trial, with assessments at three distinct time-points: pre-intervention (T1), post-intervention (one week after the end of treatment, T2), and one follow-up assessment (one month after treatment termination; T3). One independent assessor performed diagnostic evaluations to assess symptom improvement.

### 3.2 Participants

A total of seven adult patients (*M*_age_ = 38.14, *SD*_age_ = 15.27), who have been diagnosed with depression as their primary diagnosis, were included within the study (see **Table 1** for sample characteristics). One patient had to be excluded during the diagnostic interview (T1) and was referred to inpatient care, due to acute suicidality. Patients were recruited at the Christophsbad Hospital in Goeppingen as well as through various social media platforms and the official website of the Christophsbad Clinic. After an initial screening for inclusion criteria (i.e., age, primary diagnosis of depression, German proficiency), patients were invited to the laboratory for a thorough diagnostic evaluation (T1). During this appointment, patients received additional information regarding the study and provided written informed consent. Afterwards, the assessment of depressive symptomatology was conducted utilizing the Structured Clinical Interview for DSM-V Axis I (SCID-I; 37). Furthermore, the Mini-International Neuropsychiatric Interview (M.I.N.I.; 38) was utilized to determine other concomitant Axis-I diagnoses. In addition, the evaluation of Axis-II comorbidity was conducted utilizing the Structured Clinical Interview for DSM-V Axis II self-report questionnaire (SCID-5-SPQ; 37). Moreover, clinical symptomatology was evaluated using the below described self-report measurements. Lastly, a chairwork interview intake form, developed by Pugh (39) and modified by JK, was administered to generate potential treatment objectives, for the upcoming therapeutic sessions.

**Table 1:** Patient characteristics.

	Patient 1	Patient 2	Patient 3	Patient 4	Patient 5	Patient 6	Patient 7
Age	22	63	43	36	30	22	51
Sex	female	female	male	male	female	female	female
Education	high school diploma/A-levels	secondary school graduation	vocational training	master’s degree	bachelor’s degree	high school diploma/A-levels	high school diploma
Marital Status	single	married	married	married	single	single	in a relationship
Primary Diagnosis	MDD	MDD	MDD	MDD	MDD	MDD	MDD
Secondary/Comorbid Diagnoses	dysthemia	agoraphobia without panic disorder	none	GAD	none	social phobia, dysthemia	GAD, panic dirsorder
Psychotherapy history	none	none	past psychotherapy	past psychotherapy	none	past psychotherapy	past psychotherapy
Psychopharmaca	none	Sertralin Pipamperon	Sertralin Opipramol	none	none	Sertralin	Bupropion Agomelatin

GAD, Generalized Anxiety Disorder; MDD, Major Depressive Disorder.

Participants were included within the study if they met the following criteria: minimum of 18 years of age, proficiency in both written and spoken German, and meeting the diagnostic criteria for depression as outlined in the International Classification of Diseases, 10th Revision (ICD-10; 40) under the categories F32.0–F32.2 and F33.0–F33.2. Patients were excluded if they were currently receiving psychotherapeutic treatment, were using acute psychiatric medication such as benzodiazepines, or were diagnosed with severe comorbid psychiatric diseases like psychotic disorder or bipolar disorder, acute suicidality, and drug misuse or dependency. Other psychopharmacological medication intake had to be stable for four weeks before study participation. The study was approved by the Ethics Committee of the State Chamber of Physicians of Baden-Wuerttemberg, Germany (Number of Approval: F-2023–12).

### 3.3 Intervention

The cognitive behavioral chairwork intervention conducted follows a semi-structured approach and follows the model of Multiplicity of the Self outlined by Pugh (41). This semi-structured approach was chosen in order tailor the treatment session to the patients’ individual concerns and goals for the session. The implemented intervention consists of three therapeutic sessions that take place over a period of three consecutive weeks. Each session lasts approximately 90 minutes. During the first session, the chairwork intake form will be collaboratively examined with the patient, and potential topics related to self-criticism, the use of I-positions in relation to the complaint, and dialogical dysfunctions will be detected (see **Supplementary Material** for further information). During this therapeutic stage, the individual is prompted to provide a concise overview of their complaint and their objective for the therapeutic session. The psychologist will subsequently formulate a dialogical hypothesis by considering both, the information provided in the intake form and the patient’s reported complaint. Following this, the psychologist will proceed to explain and demonstrate the chairwork technique. Doing so, the therapist places two chairs across from each other. Thereinafter, the therapist stands behind each chair, while speaking from the exemplary respective I-position’s stance (e.g., the I-position of the inner critic and the anxious, fearful, or sad self), in order to explain the technique to the patient. Subsequently, the idea of center will be elucidated by illustrating how the client’s initial chair serves as a secure and inert space in which the patient can cultivate self-awareness and facilitate the (re)integration of various I-positions. This chair is designed to enhance metacognitive skills (42).

Following this preliminary introduction, the chairwork session will enter its active stage. Initially, the individual will be inquired about any apprehensions or reservations pertaining to the chairwork procedure. In the event that the patient expresses hesitations, a conversation will be initiated with the inner protector (41). Doing so, a chair that serves as the I-position of the inner protector is placed within the therapy room across from the patient. Thereinafter, the patient is instructed to sit on this chair and speak from the stance of the inner protector. The therapist then validates the protector’s underlying needs and fears, offers reassurance (e.g., to not to laugh at the patient), and asks for permission to proceed with the chairwork intervention. If the inner protector does not give permission to work with the patient, the first session’s chair dialog will be conducted with the inner protector, as working with other I-positions will not be possible otherwise. If the inner protector agrees to proceed with the intervention, the inner protector chair is placed in the background of the room in order to be distant from the intervention setting, but close enough to protect the patient if needed. Subsequently, a brief diagnostic chairwork exercise will be implemented, during which the therapist will engage in an interview with the various I-positions. Subsequently, a brief diagnostic chairwork exercise will be implemented, during which the therapist will engage in an interview with various I-positions. This exercise serves as both a warm-up activity and a means to assess the validity of the dialogical premise that has been formulated by the psychologist. The identified problem will be addressed by positioning the patient on two separate chairs, symbolizing contrasting perspectives (e.g., the inner critic and the anxious self). This arrangement will facilitate a discussion initiated by the patient, allowing for a comprehensive exploration of the conflicting inner I-positions (for a detailed description, see 41). After this stage, the patient will be encouraged to witness the enactment from a third-person perspective by standing alongside the therapist in front of the two chairs. This standing position represents a more detached and compassionate observer stance, which initiates a collaborative process with the psychologist to critically analyze the chairwork experience. Doing so, the patient is instructed to witness, summarize, and reflect on the enactment from the stance of the benevolent companion, providing his opinion from a detached stance. Subsequently, the patient will be directed to return to their original seating position (i.e., center). The patient and the therapist will then proceed to develop a concise summary of the session, followed by the formulation of significant key points and insights gained. Within the following discussion, the therapist and the patient jointly examine one to two possible solutions to the problem that were assessed during the therapy session. Additionally, the therapist and the patient will outline the necessary actions that need to be taken in order to properly resolve this issue. The solutions will be recorded on a personally created diary card, which acts as a tool to help incorporate the generated solutions into one’s daily schedule. The treatments primarily consist of standard behavioral therapy interventions, such as engaging in positive activities and cultivating positive self-talk. Finally the take-home messages will be captured by audio recording, utilizing the patient’s mobile device. Finally, in case of any remaining time, the psychologist will proceed with a role-playing exercise. During this exercise, the psychologist will assume the character of the problem or the previous solution, while the patient will embody the newly devised resolution (see **Table 2** for each patient’s main complaint, therapy goals, and first session content).

**Table 2:** Main complaint, therapy goals, and first session content.

	Patient 1	Patient 2	Patient 3	Patient 4	Patient 5	Patient 6	Patient 7
Main Complaint	Lack of drive, loss of self-esteem/finding own identity	Loss of interest and pleasure. Tiredness and lack of energy. Feelings of worth-lessness, reduced self-confidence, self-blame	Feelings of sadness, worthlessness, anger, helplessness	Trouble thinking. Feelings of anxiety, sadness and guilt. Loss of pleasure and interest. Reduced self-esteem and self-care	Reduced self-esteem, feelings of worthlessness and helplessness. Social withdrawal	Feelings of sadness and loneliness. Problems with self-acceptance and self-esteem. Anxiety of losing a relationship	Feelings of guilt and pressure to fulfill other’s needs. Sleep disturbances, worrying. Social withdrawal
Goals for Therapy	Gaining self-esteem and energy, reducing sadness and inner tension	Finding solutions for reoccurring difficulties	Communicating his needs and boundaries to his wife and standing up for them (after wife’s infidelity, he subjugates to all her wants and needs in order not to be abandoned)	Making decisions on everyday issues. Reduce escaping into taking care of others. Implementing self-care and pleasure	Increase self-care and self-confidence. Reduction of social isolation and emotional distancing	Clarification of distinct inner parts and increasing the motivation to work with them, especially the complaisant companion	Reduction of anxiety. Increase self-confidence and self-care. Improve to handle with mental stressful situations
Main Emotions during Chairwork	Anger, Fear, guilt, sadness	Fear, guilt, sadness	Fear, guilt, anger, sadness	Fear, guilt, sadness	Fear, guilt, sadness	Fear, guilt, sadness	Fear, sadness
Session 1							
*Type of Conflict*	Reducing the inner protector to be emotionally available. The inner critic undermines self-confidence and demands performance (doing something special)	Elaboration of the functionality of the inner protector and reduction to a level where the patient is emotionally reachable. Reducing the inner critic to be able to adhere to own boundaries	Pleasing his wife because he is afraid of losing her. This strategy leads to more conflicts with wife	Elaboration of the functionality of the inner protector and reduction to a level where the patient is emotionally reachable. Clarifying the origin, functionality, positive and negative con-sequences of the inner critic	Elaboration of the origin and functionality of the inner protector. Clarify the relationship with the inner critic	Elaboration of the origin and functionality of the inner protector. The protector wants the patient to avoid talking about her problems to avoid the feeling of shame	The patient’s inner protector was highly activated. Therefore, she did not have any goals for the first session
*Resolution*	Limiting the inner critic and empower the compassionate/benevolent inner part	Reducing the inner critic and increasing benevolence in case of failure	Reducing the inner pleaser and distance from the inner critic (guilt inducing part)	Reducing the inner critic, as this part causes uncertainty	Implementing the benevolent part to be able to limit the inner critic	Disempower the fear of shame by speaking openly with the therapist to achieve a corrective relationship experience	Clarification of the functionality of the inner protector and its pros and cons. The believe “not to talk about depressive symptoms protects against depressive symptoms” was identified
Goal for the upcoming week	Reading a book (limiting the inner critic). When the inner critic is active consider what the benevolent inner part would say	Note what the inner benevolent part would say as a counter-position to the inner critic	Noticing when the inner pleaser is active and solely execute every second of his suggestions	Conscious perception of the activation of the inner critic and making a note of it	Setting the alarm clock to every four hours and writing down: Which inner part is active at the moment? What would the benevolent inner part say? (Focusing on implementing this inner part)	Conscious perception of the activation of the inner critic. Collecting and writing down evidence which disempowers the inner critic	Conscious perception of the activation of the inner parts. Implement the benevolent inner part by conscious asking what would he/she say?

The following treatment sessions, namely sessions two and three, will commence with a comprehensive evaluation of the diary card (see **Supplementary Material**). The diary card evaluates challenging situations that have emerged since the previous therapeutic session. Furthermore, the patient is provided with instructions to identify significant subjective viewpoints (referred to as I-positions) and engage in introspection regarding both adaptive and maladaptive behaviors associated with the given situation, as outlined in the diary card. Finally, the patient is encouraged to generate preliminary ideas on how to conduct functional problem-solving. Upon careful examination of the diary card, the clinical psychologist will provide a concise overview of the preceding treatment session with the patient, while also identifying any unresolved questions or issues. Based on an analysis of the diary card and the review of the previous session, another problem area will be identified and addressed using the chairwork technique outlined in the preceding paragraph. Following the conclusion of the second session, the patient will once again be provided with an audio recording, along with a diary card, to be utilized throughout the forthcoming week.

### 3.4 Measurements

The Beck Depression Inventory II (BDI-II; 43) is a widely used assessment tool for measuring the severity of depressive symptoms. The self-report questionnaire consists of 21 items that evaluate symptoms related to negative mood and affect on a 4-point Likert scale (from *0* = no symptoms present, to *3* = severe symptoms present). The questionnaire demonstrates good internal consistency, as evidenced by a Cronbach’s alpha coefficient ranging from .84 to .90 (44).

The Emotion Regulation Questionnaire (SEK-27; 45) consists of 27 items which evaluate various dimensions of emotion regulation. Each item can be rated on a 5-point Likert scale, ranging from *1* = not at all, to *5* = almost always. The questionnaire comprises nine distinct subscales: clarity, bodily perception, acceptance, attention, comprehension, willingness to engage with one’s emotions, regulation, resilience, and self-support. Additionally, it is possible to create an overall score. The measurement exhibits high internal consistency, as evidenced by a Cronbach's alpha coefficient of .93 (45).

The Forms of Self-Criticizing/Reassuring Scale (FSCRS; 46) is a self-report questionnaire consisting of 22 items, which assesses self-criticism and self-reassurance on a 5-point Likert scale (from *0* = not like me at all, to *4* = extremely like me). The measurement evaluates two distinct forms of self-criticism: feelings of inadequacy and a hating oneself, accompanied by a desire to inflict harm. The measurement has high internal consistencies, Cronbach’s α = .86 –.90 for the three subscales (46).

The Self-Compassion Scale (SCS; 47) is a self-report questionnaire consisting of 26 items that aim to evaluate several dimensions of self-compassion on a 5-point Likert scale ranging from *1* = almost never, to *5* = almost always. The measurement comprises six distinct subscales that assess self-kindness, mindfulness, self-judgement, over-identification, isolation, and common humanity. In addition, it is possible to compute a comprehensive score. The measurement exhibits high internal consistency, as evidenced by a Cronbach’s α = .86 (47).

The Rosenberg Self-Esteem Scale (RSES; 48) is a widely used questionnaire that assesses self-esteem. The RSES consists of 10 items, which are rated on a 4-point Likert scale (from *0* = not at all, to *3* = very much). All ten items form an overall score. The measurement exhibits high internal consistency, as evidenced by a Cronbach’s alpha coefficient of .88 (49).

### 3.5 Acceptability, feasibility, and safety

The assessment of treatment acceptability and satisfaction was conducted using a self-developed questionnaire consisting of nine items. Visual analogue scales (ranging from 1–10) were employed to measure participants’ responses. Examples of the items include statements such as “I benefitted from the treatment” and “I would recommend this treatment to individuals facing similar issues.”

The Beck Scale for Suicidal Ideation (BSI; 50) will be utilized to monitor adverse events. The purpose of this 21-item self-report questionnaire is to evaluate an individual’s risk for suicide. The assessment encompasses evaluations for suicidal ideation, thinking, and planning, as well as other facets of attempts to commit suicide. The assessment will be conducted at all time points of examination (T1–T3). The primary five questions in the questionnaire function as a means for determining the presence of suicidal tendencies. Patients who indicate a score of two on question numbers four and/or five (“I have a strong desire to kill myself, and would not take any measures to safe myself if I would find myself in a life-threatening situation”) will receive an additional evaluation from the study psychologist (JM). If a patient appears to be at risk for suicide during any contact with the clinical psychologist, a referral for additional care will be initiated (e.g., to the psychiatric inpatient hospital; see 51 for a detailed description).

### 3.6 Data analysis

First, in line with the pre-post design, treatment effects were determined by visually inspecting data for each patient individually. This approach facilitates the assessment of each individual’s change over time and allows for an evaluation of each patient’s range and continuity of change. However, simply analyzing descriptive data may contribute to Type I error. Therefore, changes in outcome measures were also examined utilizing percentage values. Treatment response (defined as a 30% reduction in depressive symptomatology) was evaluated for each patient. Additionally, reliable change as measured by the RCI (52) was assessed for each patient using standard deviations and alpha coefficients of a previous study including a general population screened for depression using the BDI-II (53). A RCI ≥ 1.96 is indicative for a significant change. In addition, paired sample t-tests were conducted to assess changes from pre-, to post-treatment (T1-T2) and follow-up (T1-T3) for the group as a whole. Cohen’s *d* was used to evaluate pre to post and follow-up treatment effect sizes (54).

## 4 Results

### 4.1 Changes in clinical symptoms: depression and emotion regulation

**Table 3** presents scores for all patients, measurements and measurement time-points.

**Table 3:** Outcome measures and symptom reduction across chairwork intervention.

		Patient 1	Patient 2	Patient 3	Patient 4	Patient 5	Patient 6	Patient 7	Mean (*SD*)	Effect size *d^1^*
BDI-II	T1	51	29	27	45	41	30	52	39.29 (10.63)	
	T2	34	28	25	24	28	28	32	28.43 (3.55)	1.21**
	T3	28	27	22	28	28	30	41	29.14 (5.79)	1.22**
% reduction of depressive symptoms	T1-T2	33	3	7	47	32	7	39	28	
	T1-T3	45	7	19	38	32	0	21	26	
SEK-27	T1	82	98	80	78	68	97	58	80.14 (14.43)	1.02*
	T2	117	95	88	103	127	97	109	105.14 (13.55)	1.21**
	T3	108	120	91	103	129	96	76	103.29 (17.85)	
% improvement in emotion regulation	T1-T2	43	3 (decrease)	10	32	87	0	88	31	
	T1-T3	32	22	14	32	90	1 (decrease)	31	29	
FSCRS	T1	69	52	43	67	50	53	74	58.29 (11.60)	0.80*
	T2	45	40	47	56	40	58	66	50.29 (9.91)	0.70*
	T3	43	51	43	59	36	54	73	51.29 (12.31)	
SCS-D	T1	73	72	85	70	77	80	64	74.43 (6.90)	0.74*
	T2	95	81	81	78	101	81	62	82.71 (12.55)	1.00*
	T3	106	90	86	77	99	78	72	86.86 (12.39)	
RSES	T1	24	32	31	22	24	24	27	26.29 (3.86)	
	T2	35	34	30	28	35	27	31	31.43 (3.31)	1.14**
	T3	34	38	30	26	34	27	21	30.00 (5.80)	*0.64*

BDI-II, Beck Depression Inventory Second Edition; SEK-27, Emotion Regulation Questionnaire; RSCRS, Forms of Self-Criticizing/Reassuring Scale; SCS-D, Self-Compassion Scale German Version; RSES, Rosenberg Self-Esteem Scale; ^1^ Cohens d was reported for T0-T1 (T1), T1-T2 (T2) and T1-T3 (T3); *, significant on the.05 level; **, significant on the.01 level.

**Figure 1:**
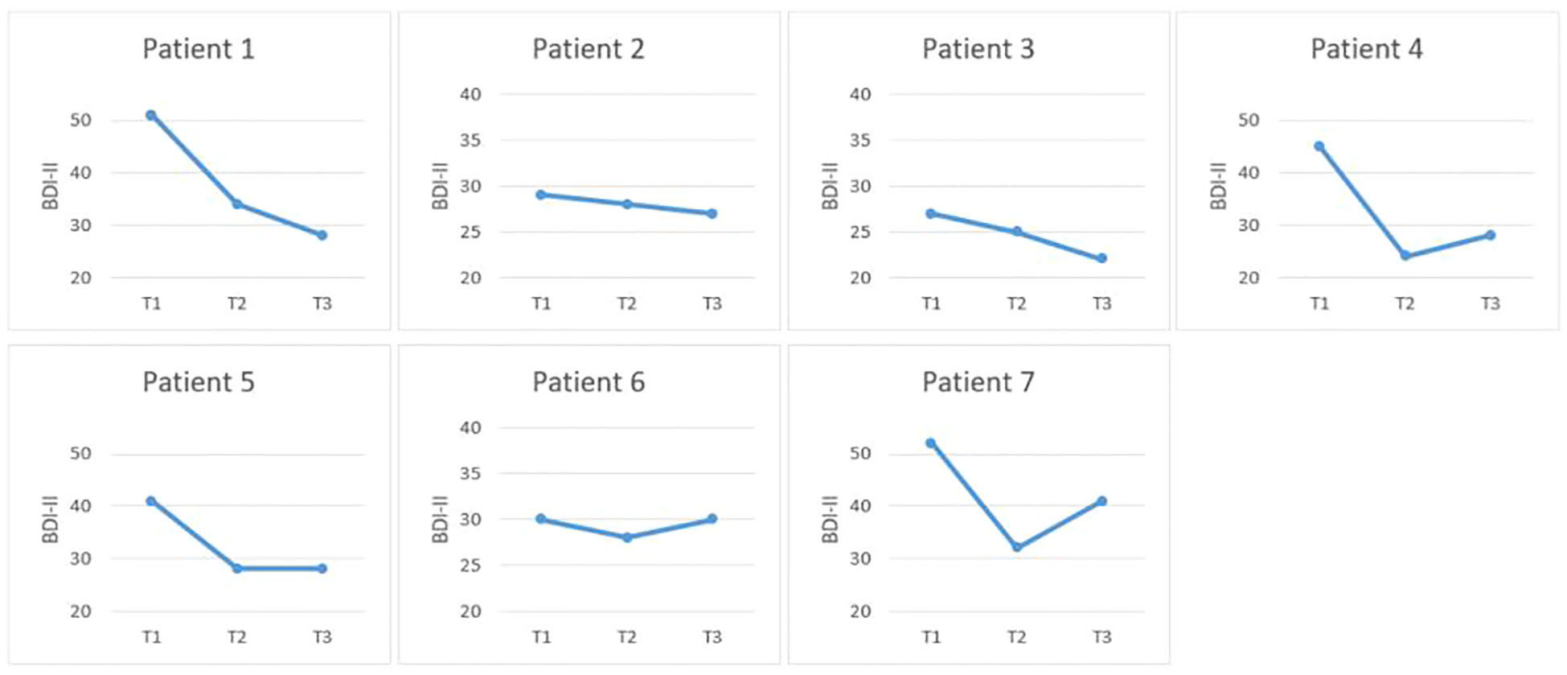
Symptomatic improvement in depressive symptoms from T1 to T3 (BDI-II).

Looking at depressive symptoms as assessed by the BDI-II, there was a significant 28% decrease in depressiveness for the group as a whole one week after treatment termination, *t*(6) = 3.20, *p* ≤ 0.01, *d* = 1.21, as well as a 26% reduction in depressive symptoms, *t*(6) = 3.22, *p* ≤ 0.01, *d* = 1.05, one month after treatment termination, demonstrating that gains were maintained during the follow-up period. Four out of seven patients met criteria for being treatment responders (≥ 30% reduction in depressive symptoms at follow-up) whereby all four of these patients (1, 4, 5, 7) revealed RCIs greater than 1.96 one week- as well as one month after the intervention, indicating significant changes in depressive symptoms. The remaining three patients (patient 2, 3, 6) did not display clinical change according to the RCI at one month follow-up, however, patient 2 displayed a 7%, and patient 3 a 19% reduction in depressive symptoms as compared to the baseline measurement time-point. Nevertheless, patient 6 did not show any changes in depressive symptoms at follow-up (0%; see **Figure 1** for symptomatic changes by patient).

With respect to emotion regulation (SEK-27), there was a significant 31% increase in adaptive emotion regulation one week after treatment termination, *t*(6) = −2.69, *p* ≤.05, *d* = 1.02. Moreover, there was a significant 29% increase in adaptive emotion regulation one month after treatment termination, *t*(6) = −3.20, *p* ≤ 0.01, *d* = 1.21, for the group as a whole, demonstrating that gains were maintained during the follow-up period. All patients, except for patient 6 demonstrated improvements in emotion regulation at follow up.

### 4.2 Changes in self-reassuring/self-criticizing, self-compassion, and self-esteem

Regarding self-reassuring/self-criticizing (FSCRS), there was a significant 14% decrease in dysfunctional symptoms, indicating that self-reassuring tendencies increased, while self-criticizing self-verbalizations decreased one week after treatment termination, *t*(6) = 2.12, *p* ≤ 0.05, *d* = 0.80, and a significant 12% decrease in the latter symptoms one month after treatment termination, *t*(6) = 1.86, *p* ≤ .05, *d* = 0.70, which displays that gains were maintained during the follow-up period.

With respect to self-compassion (SCS-D), there was a significant 11% increase in self-compassion from baseline to post-treatment, *t*(6) = −1.97, *p* ≤ 0.05, *d* = 0.74 for the group as a whole. Furthermore, self-compassion significantly increased about 17% one month after treatment termination, *t*(6) = −2.64, *p* ≤ 0.05, *d* = 1.00, demonstrating that further gains in symptom improvement were achieved during the follow-up period.

Looking at to self-esteem (RSES), there was a significant 20% increase in self-esteem one week post-intervention as compared to baseline, *t*(6) = −3.01, *p* ≤ .01, *d* = 1.14. Additionally, there was a trend 14% increase in self-esteem one month after treatment termination, *t*(6) = −2.64, *p* = .06, *d* = 0.64, showing that gains could partially be maintained at follow-up.

### 4.3 Acceptability, feasibility, and safety

At the initial screening, 16 patients were briefly evaluated, with eight patients meeting inclusion criteria. During the diagnostic assessment (T1), one patient had to be excluded and referred for inpatient care, due to acute suicidality. The remaining patients completed all diagnostic assessment time-points as well as all three therapy sessions.

Patient 7 indicated suicidal thoughts at T2 as well as at T3. According to the protocol, the study therapist (JM) contacted the patient both times by phone to evaluate the risk for committing suicide. At T2 the patient indicated that she was diagnosed with a severe neurological disorder, which has caused the worsening of her psychological symptoms. Despite having suicidal thoughts, patient 7 was committed to staying alive and not act upon her suicidal thoughts. The study therapist recommended patient 7 for supportive outpatient care within our clinic at T3.

**Figure 2:**
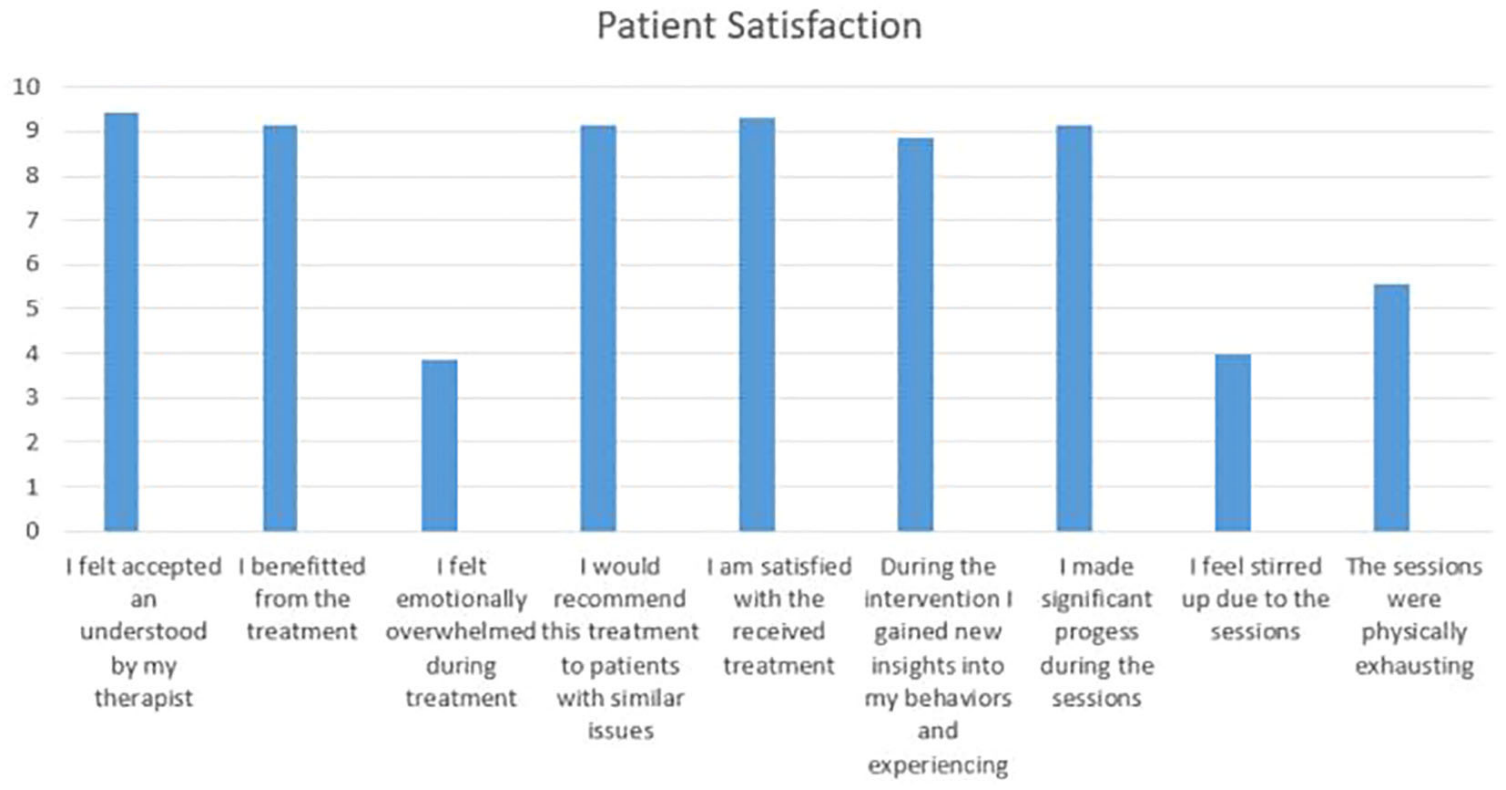
Patient satisfaction.

Overall, all patients were highly satisfied with the chairwork short-intervention (*M* = 8.29, *SD* = 0.85). Detailed responses to the items within the patient satisfaction questionnaire can be found in **Figure 2**.

## 5 Discussion

The aim of the present study was to examine the efficacy, feasibility, and safety of a three-session short-intervention implementing CBT based chairwork to treat symptoms associated with self-criticism in patients diagnosed with depression. Moreover, the here presented findings shall improve the understanding of the functionality of specific treatment techniques for targeted psychological symptoms. The findings of the current study indicate that the implemented intervention was very well accepted amongst the included patients. Solely one patient had to be excluded during the diagnostic interview (T1) due to suicidality. The remaining seven patients completed all therapeutic sessions and assessments. Moreover, all patients were highly satisfied with the treatment and indicated that they benefitted from the three therapeutic sessions. Additionally, the implemented treatment was safe for all included patients. Solely one patient indicated suicidal thoughts at both post-intervention assessment time-points, which were due to a newly diagnosed neurological disorder, rather than the treatment received.

Looking at treatment outcomes, our results provide initial evidence for the efficacy of the three-session chairwork intervention. Specifically, there was a significant and lasting improvement in depressive symptoms (BDI-II) for the group as a whole across both follow-up assessments, revealing large effect sizes. This finding is aligning with previous studies, which demonstrated the beneficial effects of chairwork on symptoms of depression (29, 36). Furthermore, dysfunctional emotion regulation (SEK-27) improved across the investigated group from pre-treatment to post-treatment as well as follow-up significantly, with large effect sizes. This finding is particularly important, as past studies have shown that improving emotional processing in individuals diagnosed with depression leads to better therapeutic outcomes than merely focusing on alterations on a cognitive level (17–19). However, the present study did not compare standard CBT to emotion focused CBT using chairwork. Therefore, no definite answer can be given as to whether cognitive or emotion focused approaches are better suited to improve emotional processing. Future research could hence focus on comparing both approaches when targeting emotional processes. Lastly, looking at individual scores regarding depressive symptoms and improvement in emotion regulation strategies, all but one patient (patient 6) showed symptomatic improvement. When contacting this patient about her symptoms, she explained that a major life event has occurred (i.e., loss of an important relationship), which has led to continued feelings of depression. Interestingly, our findings further align with common cognitive models of depression, which postulate that the beginning, maintenance, and occurrence of depressive disorders is related to distorted information processing routed in negative schemas (4). While standard CBT techniques focus on cognitive restructuring or Socratic questioning, oftentimes resulting in intellectual, rather than affective change (7), chairwork is capable of directly targeting “hot” cognitions (55, 56), therefore resulting in emotional, as well as intellectual change.

Regarding self-compassion (SCS-D), there was a significant improvement from pre-treatment to post-treatment for the group as a whole, demonstrating a large effect size. Moreover, symptoms continuously improved up to the one-month follow-up assessment, showing that further gains were achieved across the follow-up period. Similar results were found regarding self-criticism/self-reassuring (FSCRS): Scores decreased from pre-treatment to post-treatment and remained comparably stable at follow-up, with large effect sizes at both pre-post comparison time-points. These findings demonstrate that emotion-focused chairwork targeting self-criticism is capable of increasing self-compassion, while decreasing self-criticism at the same time. It is interesting that in contrast to all other included patients, patient 6 showed a decrease in self-compassion and an increase in self-criticism, while there was no improvement in either emotion dysregulation or depressive symptoms. Therefore, it could be hypothesized that improvement in depressive symptoms solely takes place if similar improvements in self-compassion/self-criticism are achieved. This notion is partially supported by previous studies, which demonstrated that patients diagnosed with depression reported less self-compassion (57, 58). Moreover, self-compassion also functioned as a protective factor in non-clinical individuals for developing symptoms of depression (59). However, as the current study solely focused on symptomatic pre-post improvement, further research is needed to detangle the circumstances under which depressive symptomatology can be improved. Lastly, self-esteem improved from pre-treatment to post-treatment for the group as a whole, revealing a large effect size. However, while this improvement remained comparably stable up until the follow-up assessment, this result remained no longer significant. When looking at the individual patient’s results, it becomes apparent that most patients experienced a steep incline in self-esteem one week post-treatment. Nevertheless, these results declined across several patients from post-treatment to one-month follow up. This finding could be due to the fact that self-esteem is routed in several distinct facets, such as self-acceptance, self-assertiveness, self-responsibility, or personal integrity (60). While the current intervention might have targeted some of the included aspects, which might have led to short-term symptom improvement, the treatment might not have made a significant and lasting impact across all facets of self-esteem. Furthermore, recent research has begun to alter and update the model of the inner critic towards a two-critic model (e.g., 61, 62). Within this updated model, the inner critic is compartmentalized into two distinct critics, with one inner critic symbolizing the internalization of abusive experiences (i.e., the internalized abuser), while the other critic is defined as a coping mode, which was developed in order to help the individual survive (i.e., the protector critic). This development is very important for future research, as the distinction between the two critic modes and associated tailored treatments could possibly further improve self-compassion.

Taken together, the implemented treatment appears to have good efficacy, feasibility, and safety when treating symptoms associated self-criticism in patients with depression. Six out of seven patients experienced a decline in several clinical symptoms at post-treatment as well as one months after the intervention. Solely one of the included patients reported no improvement regarding her symptoms in comparison to pre-treatment scores, which was due to a significant life-event. Furthermore, the conducted intervention was highly accepted by all patients, who were very satisfied with the received treatment.

While the current study has several strengths, such as the use of a semi-structured short-intervention treatment allowing for high treatment replicability, as well as the implementation of a one-month follow-up assessment to evaluate long-term treatment outcomes, it does not come without its limitations. First, the current intervention was conducted within an outpatient setting. Thus, results might not transfer to inpatient treatment programs. Second, the implemented study solely included an intervention group without including an active or passive control group. Therefore, no conclusions can be drawn regarding the effectiveness of the implemented treatment in comparison to other treatment programs. Third, other unspecific factors, such as the patient’s expectations or the patient-therapist relationship need to be considered when interpreting the presented results. Fourth, while we recorded adverse events such as suicidality, emotional arousal, and physical fatigue after treatment termination within the patient satisfaction questionnaire, we did not evaluate other adverse events following the intervention, such as physical and psychological symptoms (e.g., headaches, physical tension, mood elevation). As up to 53% of patients diagnosed with depression, who are undergoing psychotherapy, report adverse treatment effects (63), it would be important to routinely assess those symptoms after each treatment session in order to swiftly determine adverse effects and provide appropriate interventions. Fifth, while we assessed if the included patients underwent psychotherapeutic treatment in the past, we did neither specify the treatment received, nor the duration of treatment, nor the psychotherapeutic setting. Lastly, the sample size of the current study was comparably small, resulting in limited generalizability of the presented findings. To overcome the described drawbacks, future research could expand onto the presented findings by conducting a large-scale randomized controlled trial (RCT), which includes the described missing information, to evaluate the effectiveness of the newly developed chairwork treatment.

## Data availability statement

The raw data supporting the conclusions of this article will be made available by the authors upon reasonable request.

## Ethics statement

The studies involving humans were approved by Ethics Committee of the State Chamber of Physicians of Baden-Wuerttemberg, Germany. The studies were conducted in accordance with the local legislation and institutional requirements. The participants provided their written informed consent to participate in this study. Written informed consent was obtained from the individual(s) for the publication of any potentially identifiable images or data included in this article.

## Author contributions

JK: Conceptualization, Data curation, Formal analysis, Methodology, Project administration, Resources, Supervision, Visualization, Writing – original draft, Writing – review & editing. JM: Conceptualization, Data curation, Investigation, Methodology, Writing – review & editing. ZS-V: Funding acquisition, Resources, Supervision, Writing – review & editing.

## References

[1] APA. Diagnostic and statistical manual of mental disorders: DSM-5. (2013). doi: 10.1176/appi.books 24413388

[2] Greenberg LS Watson JC. Emotion-focused therapy for depression. Washington DC, Washington, USA: American Psychological Association (2006). doi: 10.1037/11286-000

[3] Demiralp E Thompson RJ Mata J Jaeggi SM Buschkuehl M Barrett LF. Feeling blue or turquoise? Emotional differentiation in major depressive disorder. Psychol Sci. (2012) 23:1410–6. doi: 10.1177/0956797612444903 PMC4004625 23070307

[4] Beck JS. Cognitive Therapy: basics and beyond. New York, NY, US: The Guilford Press (1995).

[5] Waltman SH Codd RT III McFarr LM Moore BA. Socratic questioning for therapists and counselors: Learn how to think and intervene like a cognitive behavior therapist. Abingdon, Oxfordshire: Routledge TS - RIS (2020). doi: 10.4324/9780429320392

[6] Beck AT. Cognitive therapy and the emotional disorders. New York, USA: Penguin TS (1979).

[7] Goldfried MR. Evidence-based treatment and cognitive-affective-relational-behavior-therapy. Psychotherapy. (2013) 50:376. doi: 10.1037/a0032158 24000855

[8] Westen D Morrison K. A multidimensional meta-analysis of treatments for depression, panic, and generalized anxiety disorder: an empirical examination of the status of empirically supported therapies. J Consult Clin Psychol. (2001) 69:875. doi: 10.1037//0022-006X.69.6.875 11777114

[9] Moreno JL. Psychodrama. New York: Beacon House (1948). 1 M4-Ci.

[10] Pearls F. The gestalt approch and eye witness to therapy. New York, NY, US: Science & Behavior Books (1973).

[11] Greenberg LS. Emotion-focused therapy. Washington, DC, US: American Psychological Association (2011).

[12] Young JE Klosko JS Weishaar ME. Schema therapy: A practicioner´s guide. 1st Edn. New York, USA: Guilford Press (2006).

[13] Jacob GA Arntz A. Schema therapy for personality disorders—A review. Int J Cognit Ther. (2013) 6:171–85. doi: 10.1521/ijct.2013.6.2.171

[14] Pugh M. Chairwork in cognitive behavioural therapy: A narrative review. Cognit Ther Res. (2017) 41:16–30. doi: 10.1007/s10608-016-9805-x

[15] Pugh M. Cognitive behavioural chairwork. Int J Cognit Ther. (2018) 11:100–16. doi: 10.1007/s41811-018-0001-5

[16] Pugh M. Cognitive behavioural chairwork: Distinctive features. Oxon, UK: Routledge (2019). doi: 10.4324/9780429023927

[17] Hunt MG. The only way out is through: Emotional processing and recovery after a depressing life event. Behav Res Ther. (1998) 36:361–84. doi: 10.1016/S0005-7967(98)00017-5 9670599

[18] Hayes AM. Facilitating emotional processing in depression: the application of exposure principles. Curr Opin Psychol. (2015) 4:61–6. doi: 10.1016/j.copsyc.2015.03.032 PMC4410732 25932464

[19] Pinheiro P Gonçalves MM Sousa I Salgado J. What is the effect of emotional processing on depression? A longitudinal study. Psychother Res. (2021) 31:507–19. doi: 10.1080/10503307.2020.1781951 32558621

[20] Kellogg S. Dialogical encounters: contemporary perspectives on" Chairwork". Psychotherapy. Psychotherapy: Theory Research Practice Training. (2004) 41:310. doi: 10.1037/0033-3204.41.3.310

[21] Dugas MJ Robichaud M. Cognitive-behavioral therapy for generalized anxiety disorder: From science to practice. New York: Taylor & Francis (2007).

[22] Arntz A Weertman A. Treatment of childhood memories: Theory and practice. Behav Res Ther. (1999) 37:715–40. doi: 10.1016/S0005-7967(98)00173-9 10452174

[23] Beck AT Freeman A Davis DD. Cognitive therapy of personality disorders. 2nd Edn. New York: Guilford Press (2004).

[24] Dolhanty J Greenberg LS. Emotion-focused therapy in the treatment of eating disorders. Eur Psychother. (2007) 7:97–116.

[25] Kellogg S. Chairwork psychotherapy: using the four dialogues in the treatment of trauma. New York City Cognitive Therapy Association Newsletter (2023).

[26] Gilbert P Irons C. “Focused therapies and compassionate mind training for shame and self-attacking,”. In: Compassion. Abingdon, Oxfordshire: Routledge (2005). p. 263–325.

[27] Gilbert P. Compassion focused therapy: Distinctive features. Abingdon, Oxfordshire: Routledge (2010). doi: 10.4324/9780203851197

[28] Bell T Montague J Elander J Gilbert P. A definite feel-it moment”: Embodiment, externalisation and emotion during chair-work in compassion-focused therapy. Couns Psychother Res. (2020) 20:143–53. doi: 10.1002/capr.12248

[29] Shahar B Carlin ER Engle DE Hegde J Szepsenwol O Arkowitz H. A pilot investigation of emotion-focused two-chair dialogue intervention for self-criticism. Clin Psychol Psychother. (2012) 19:496–507. doi: 10.1002/cpp.762 21710579

[30] Oliveira IR Powell VB Wenzel A Caldas M Seixas C Almeida C. Efficacy of the trial-based thought record, a new cognitive therapy strategy designed to change core beliefs, in social phobia. J Clin Pharm Ther. (2012) 37:328–34. doi: 10.1111/jcp.2012.37.issue-3 21955037

[31] Oliveira IR. Trial-Based Thought Record (TBTR): preliminary data on a strategy to deal with core beliefs by combining sentence reversion and the use of analogy with a judicial process. Braz J Psychiatry. (2008) 30:12–8. doi: 10.1590/S1516-44462008000100003 18373017

[32] Oliveira IR Hemmany C Powell VB Bonfim TD Duran É.P Novais N. Trial-based psychotherapy and the efficacy of trial-based thought record in changing unhelpful core beliefs and reducing self-criticism. CNS Spectr. (2012) 17:16–23. doi: 10.1017/S1092852912000399 22790114

[33] Neff KD Kirkpatrick KL Rude SS. Self-compassion and adaptive psychological functioning. J Res Pers. (2007) 41:139–54. doi: 10.1016/j.jrp.2006.03.004

[34] Kramer U Pascual-Leone A. The role of maladaptive anger in self-criticism: A quasi-experimental study on emotional processes. Couns Psychol Q. (2016) 29:311–33. doi: 10.1080/09515070.2015.1090395

[35] Powell VB Oliveira OH Seixas C Almeida C Grangeon MC Caldas M. Changing core beliefs with trial-based cognitive therapy may improve quality of life in social phobia: a randomized study. Braz J Psychiatry. (2013) 35:243–7. doi: 10.1590/1516-4446-2012-0863 24142084

[36] Stiegler JR Binder P-E Hjeltnes A Stige SH Schanche E. ‘It’s heavy, intense, horrendous and nice’: clients’ experiences in two-chair dialogues. Person-Centered Experiential Psychotherapies. (2018) 17:139–59. doi: 10.1080/14779757.2018.1472138

[37] First MB Williams JBW Karg RS Spitzer RL. Structured clinical interview for DSM-5 disorders, clinician version (SCID-5-CV). Arlington, VA: American Psychiatric Association (2016).

[38] Sheehan DV Lecrubier Y Sheehan KH Amorim P Janavs J Weiller E. The Mini-International Neuropsychiatric Interview (M.I.N.I.): The development and validation of a structured diagnostic psychiatric interview for DSM-IV and ICD-10. J Clin Psychiatry. (1998) 59:22–33. 9881538

[39] Pugh M. SSC-intake. Available online at: https://chairwork.co.uk/resources/.

[40] WHO ICD-10. International statistical classification of diseases and related health problems, 10th revision (ICD-10). Geneva, Switzerland: World Health Organization (2016).

[41] Pugh M. Single-session chairwork: overview and case illustration of brief dialogical psychotherapy. Br J Guid Counc. (2021), 1–19. doi: 10.1080/03069885.2021.1984395

[42] Pugh M. Single-session chairwork: overview and case illustration of brief dialogical psychotherapy. Br J Guidance and Counselling. (2021). doi: 10.1080/03069885.2021.1984395

[43] Beck AT Steer RA Brown GK. Manual for the beck depression inventory-II. San Antonio, TX: Psychological Corporation (1996). doi: 10.1037/t00742-000

[44] Kühner C Bürger C Keller F Hautzinger M. Reliabilität und Validität des revidierten Beck-Depressionsinventars (BDI-II). Reliability and validity of the Revised Beck Depression Inventory (BDI-II). Nervenarzt. (2007) 78:651–6. doi: 10.1007/s00115-006-2098-7 16832698

[45] Berking M Znoj H. Entwicklung und Validierng eines Fragebogen zur standardisierten Selbsteinschätzung emotionaler Kompetenzen (SEK-27). Z für Psychiatrie Psychol und Psycotherapie. (2008) 56:141–53. doi: 10.1024/1661-4747.56.2.141

[46] Gilbert P Clarke M Hempel S Miles JNV Irons C. Criticizing and reassuring oneself: An exploration of forms, styles and reasons in female students. Br J Clin Psychol. (2004) 43:31–50. doi: 10.1348/014466504772812959 15005905

[47] Neff KD. The development and validation of a scale to measure self-compassion. Self identity. (2003) 2:223–50. doi: 10.1080/15298860309027

[48] Rosenberg M. Rosenberg self-esteem scale. J Relig Health. (1965). doi: 10.1037/t01038-000

[49] Castilho P Pinto-Gouveia J Duarte J. Evaluating the multifactor structure of the long and short versions of the self-compassion scale in a clinical sample. J Clin Psychol. (2015) 71:856–70. doi: 10.1002/jclp.2015.71.issue-9 25907562

[50] Beck AT Steer RA. Manual for the Beck scale for suicide ideation. San Antonio TX: psychol Corporation. (1991) 63.

[51] Kroener J Mahler J Sosic-Vasic Z. Addressing self-criticism in depression using CBT-based emotion-focused chairwork: study protocol of a randomised controlled trial. BMJ Open. (2023) 13:e073128. doi: 10.1136/bmjopen-2023-073128 PMC10619115 37899154

[52] Jacobson NS Truax P. Clinical significance: a statistical approach to defining meaningful change in psychotherapy research. J Consult Clin Psychol. (1991) 59:12–9. doi: 10.1037//0022-006X.59.1.12 2002127

[53] Nuevo R Lehtinen V Reyna-Liberato PM Ayuso-Mateos JL. Usefulness of the Beck Depression Inventory as a screening method for depression among the general population of Finland. Scand J Public Health. (2009) 37:28–34. doi: 10.1177/1403494808097169 19141552

[54] Rosenthal R Cooper H Hedges L. Parametric measures of effect size. Handb Res synthesis. (1994) 621:231–44.

[55] Goldfried MR. Application of rational restructuring to anxiety disorders. Couns Psychol. (1988) 16:50–68. doi: 10.1177/0011000088161004

[56] Samoilov A Goldfried MR. Role of emotion in cognitive-behavior therapy. Clin Psychology: Sci Pract. (2000) 7:373. doi: 10.1093/clipsy.7.4.373

[57] Krieger T Altenstein D Baettig I Doerig N Holtforth MG. Self-compassion in depression: associations with depressive symptoms, rumination, and avoidance in depressed outpatients. Behav Ther. (2013) 44:501–13. doi: 10.1016/j.beth.2013.04.004 23768676

[58] de Souza LK Policarpo D Hutz CS. Self-compassion and symptoms of stress, anxiety, and depression. Trends Psychol. (2020) 28:85–98. doi: 10.1007/s43076-020-00018-2

[59] Raes F. The effect of self-compassion on the development of depression symptoms in a non-clinical sample. Mindfulness (N Y). (2011) 2:33–6. doi: 10.1007/s12671-011-0040-y

[60] Branden N. Six pillars of self-esteem: the definitive work on self-esteem by the leading pioneer in the field. Penguin Random House, New York, USA: Bantam (1995).

[61] Kellogg S Garcia Torres A. Toward a chairwork psychotherapy: Using the four dialogues for healing and transformation. Pract Innov. (2021) 6:171–80. doi: 10.1037/pri0000149

[62] Brockman RN Simpson S Hayes C van der Wijngaart R Smout M. Cambridge guide to schema therapy. Cambridge: Cambridge University Press (2023). doi: 10.1017/9781108918145

[63] Moritz S Nestoriuc Y Rief W Klein JP Jelinek L Peth J. It can’t hurt, right? Adverse effects of psychotherapy in patients with depression. Eur Arch Psychiatry Clin Neurosci. (2019) 269:577–86. doi: 10.1007/s00406-018-0931-1 30088072

