# Supplementary material for: Working with the inner critic in patients with depression using chairwork: a pilot study
Source: Front Psychiatry. 2024 Jul 1;15:1397925. doi: 10.3389/fpsyt.2024.1397925 (PMC11248432; doi:10.3389/fpsyt.2024.1397925)
Supplement: Supplementary file 1 [file DataSheet_1.zip › Supplement 1.DOCX]

**Chairwork Interview**

The following questions are designed to clarify the focus of the chairwork intervention.

1. **What issue/problem would you like to discuss within the upcoming 3 therapy sessions using chair work?** (Is there a situation in which you were particularly critical of yourself?)
2. **In which situations/moments does the problem manifest itself? To what extent does the problem affect your everyday life?**
3. **To what extent does this problem affect people around you?**
4. **Which background information relating to the problem is worth for me to know** (e.g. experiences with important people in your life, difficult life situations or experiences)?
5. **Do you have any idea how this problem came about?** (How long has the problem existed? When did it first occur? Also: When did the depressive symptoms begin? When did it occur for the first time?)
6. **What feelings and emotions are commonly associated with this problem?**
7. **What thoughts are commonly associated with this problem?** (What does the problem say about you as a person?)
8. **How do you behave/what do you do when the problem occurs?**
9. **What would be different in your life if the problem would be gone tomorrow?**
10. **What have you tried so far to deal with or solve the problem? What has been helpful? What has been less helpful?**
11. **If the following therapy sessions could help you to take a step towards solving your problem, what could this step look like?**
12. **If the therapeutic sessions would have helped you to take a step towards solving your problem: What would have changed?**
13. **What strengths or personal resources do you have in order to deal with this problem? *If you are unsure: think about how people who know you very well would answer this question***
14. **Is there anything else you think would be important to discuss before the upcoming therapy sessions?** (E.g. information about your background, origin, sexual orientation, gender identity, culture, physical health, etc.)?
15. **Is there a particular chair dialog that you think would be helpful? E.g. with a specific person, an inner part or something else?**
16. **Do you have any experience of therapeutically working with chairs in the past?**
