# Supplementary material for: Working with the inner critic in patients with depression using chairwork: a pilot study
Source: Front Psychiatry. 2024 Jul 1;15:1397925. doi: 10.3389/fpsyt.2024.1397925 (PMC11248432; doi:10.3389/fpsyt.2024.1397925)
Supplement: Supplementary file 1 [file DataSheet_1.zip › Supplement 2.DOCX]

**Diary Card – Part 1**

Dear participant,

This dairy card is intended to assist you to transfer the insights gained during therapy to your everyday life. Please complete the questions below, which are related to your last therapy session.

| **What was your take home message from the last therapy session? Which solutions did you develop within today´s therapeutic session?** |
| --- |
| **What steps will you take during the upcoming week to further solve your problem?** |

**Diary Card – Part 2**

**Situations protocol**

| **Triggering Situation** | **I-position A**  **(Thoughts, emotions, behavioral impulses)**  **Name:** | **I-position B**  **(Thoughts, emotions, behavioral impulses)**  **Name:** | **I-position C**  **(Thoughts, emotions, behavioral impulses)**  **Name:** | **What did I actually do?**  **What part of my behavior was appropriate/helpful?**  **What part of my behavior made the situation worse/was problematic?**  **What could I have done instead?**  **How could I contribute to solving the problem?**  **What would I have needed in the situation?** |
| --- | --- | --- | --- | --- |
|  |  |  |  |  |
| **That's what my benevolent companion would tell me today:** | | | | |
